# An innovative approach for testing bioinformatics programs using metamorphic testing

**DOI:** 10.1186/1471-2105-10-24

**Published:** 2009-01-19

**Authors:** Tsong Yueh Chen, Joshua WK Ho, Huai Liu, Xiaoyuan Xie

**Affiliations:** 1 Centre for Software Analysis and Testing, Swinburne University of Technology, Hawthorn, VIC 3122, Australia; 2 School of Information Technologies, The University of Sydney, Sydney, NSW 2006, Australia; 3 NICTA, Australian Technology Park, Eveleigh, NSW 2015, Australia

## Abstract

**Background:**

Recent advances in experimental and computational technologies have fueled the development of many sophisticated bioinformatics programs. The correctness of such programs is crucial as incorrectly computed results may lead to wrong biological conclusion or misguide downstream experimentation. Common software testing procedures involve executing the target program with a set of test inputs and then verifying the correctness of the test outputs. However, due to the complexity of many bioinformatics programs, it is often difficult to verify the correctness of the test outputs. Therefore our ability to perform systematic software testing is greatly hindered.

**Results:**

We propose to use a novel software testing technique, metamorphic testing (MT), to test a range of bioinformatics programs. Instead of requiring a mechanism to verify whether an individual test output is correct, the MT technique verifies whether a pair of test outputs conform to a set of domain specific properties, called metamorphic relations (MRs), thus greatly increases the number and variety of test cases that can be applied. To demonstrate how MT is used in practice, we applied MT to test two open-source bioinformatics programs, namely GNLab and SeqMap. In particular we show that MT is simple to implement, and is effective in detecting faults in a real-life program and some artificially fault-seeded programs. Further, we discuss how MT can be applied to test programs from various domains of bioinformatics.

**Conclusion:**

This paper describes the application of a simple, effective and automated technique to systematically test a range of bioinformatics programs. We show how MT can be implemented in practice through two real-life case studies. Since many bioinformatics programs, particularly those for large scale simulation and data analysis, are hard to test systematically, their developers may benefit from using MT as part of the testing strategy. Therefore our work represents a significant step towards software reliability in bioinformatics.

## Background

### Software testing issues in bioinformatics

In this post-genomic era, we are experiencing an explosion of biological data in terms of quantity and variety. To date, a lot of bioinformatics programs have been, and are continuously being, developed to support the analysis of these data. Most of the research effort in the bioinformatics community focuses on developing advanced computational and statistical methods to support these tools. However, very little work has been reported on how to systematically and effectively test these programs. Clearly the correctness of such programs is just as important as using the best algorithm, as incorrectly computed results may lead to wrong biological conclusion, and subsequently misguide downstream experiments.

Currently, only a small amount of work has been devoted to software engineering in bioinformatics [1,2]. Further, their main focus is on the management of large and complex biological datasets, whereas issues related to the correctness of bioinformatics software is largely ignored.

By its very nature, many bioinformatics programs are developed to organize and to analyze large and complex biological datasets. Many of these programs involve (1) processing large amount of data, and (2) invoking complex processing procedures to extract useful information. In particular, due to the rapid accumulation of high-throughput datasets and the increasing focus on systems-level biological modeling, the size and complexity of bioinformatics programs are growing rapidly. This poses a great challenge in developing a good testing strategy to ensure the reliability of the software implementation.

Software testing involves defining test objectives, selecting some inputs of the software under test as *test cases*, executing the software with these test cases, and verifying testing results [3]. A good testing strategy should actively reveal as many faults as possible using a selected set of test cases. To achieve this goal, many techniques have been developed to guide how to generate good test objectives and select good test cases. Some commonly used testing techniques include random testing, domain testing, control-flow testing, data-flow testing, and so on [3,4]. However, many of these techniques implicitly assume that there is a means to verify whether the testing result is correct, which is not necessarily the case in many practical situations. In software testing, an *oracle *is a mechanism to decide if the output of the target program is correct given any possible input. When a test oracle exists, we can apply a large number and variety of test cases to test a program since the correctness of the output can be verified using the oracle. Without a tangible oracle, the choice of test cases is greatly limited to those *special test cases *[5] where the expected outputs are known or there exists a way to easily verify the correctness of the testing results. In particular, an *oracle problem *is said to exist when : (1) "there does not exist an oracle" or (2) "it is theoretically possible, but practically too difficult to determine the correct output" [6].

The existence of a tangible oracle is essential when performing systematic program testing. Since an oracle is a systematic mechanism to verify the testing outputs for all possible program inputs, the presence of an oracle enables a testing strategy to select diverse inputs for testing in a systematic manner. In practice, it is possible to test programs using some simple or special test cases that are incrementally developed along the software development process. However, these test cases most likely only constitute a small portion of the whole input domain, and therefore cannot substitute a systematic oracle.

The oracle problem is a challenging topic for testing programs. Unfortunately, many bioinformatics programs has the oracle problem as it is usually very difficult to construct a practical oracle to verify the results of all possible inputs of a program. Since most commonly used testing techniques (such as random testing, domain testing, and so on) assume the existence of an oracle, they are not appropriate for testing bioinformatics programs of which the oracles do not exist. This problem is particularly relevant in the area of biological network simulation. The challenge of objective testing of both deterministic and stochastic simulators has been realized by the SBML community [7,8]. To test the reliability of a new SBML capable simulator, the current practice involves executing it with multiple existing simulators on some well studied input models and compare the consistency of the simulation results [7]. Such a method is called *N-version programming *[9]. Although such effort seems to be satisfactory, it is not universally applicable since it relies on the availability of multiple implementations of a program with the same input/output structure, which is often hard to obtain. Further, it is difficult to judge what is the correct output when different simulators generate very different results. It has been noted that " [a]s no authoritative result set exists, it is hard to devise a metric based on the simulation results, that would tell us whether a given simulation result is 'correct' or not" [7]. Visual inspection of the simulation results has been suggested to help us distinguish unreasonable results from reasonable ones. Nonetheless, this approach is not automatable, subjective, and requires the tester to have expert knowledge of the underlying algorithm. Therefore we need an alternative testing strategy to address the oracle problem.

### Metamorphic testing

*Metamorphic testing *(MT) [5,6,10-13] is an innovative testing approach to alleviate the oracle problem. Instead of using the traditional test oracle, MT uses some problem domain specific properties, namely *metamorphic relations *(MRs), to verify the testing outputs. The end-users, together with the testers or program developers, first need to identify some properties of the software under test. Then, MRs can be derived according to these properties. Some test cases (namely *source test cases *in the context of MT) can be selected according to some traditional testing techniques. Further test cases (namely *follow-up test cases *in the context of MT) can be generated based on the source test cases and according to the MRs. All test cases are executed, and then the outputs of the source and follow-up test cases are checked against the MRs. If any pair of source and follow-up test cases violates (that is, does not satisfy) their corresponding MR, the tester can say that a failure is detected and hence conclude that the program has bugs.

Chen *et al*. [10] have used some examples to illustrate how to use the MT technique. One example is to test a program that searches for the shortest path between any two nodes in an undirected graph. Given a weighted graph *G*, a source node *x*, and a destination node *y *in *G*, the target program is to output the shortest path and the shortest distance, *d*(*x*, *y*, *G*), from *x *to *y*. In this problem, a practically feasible test oracle does not exist due to the combinatorially large number of possible paths between *x *and *y*. Therefore testing such a program is difficult due to the oracle problem. Using the MT technique, we can define a number of MRs based on some well-known properties in graph theory. Here we use two such properties as examples: *d*(*x*, *y*, *G*) = *d*(*y*, *x*, *G*), and *d*(*x*, *y*, *G*) = *d*(*x*, *w*, *G*) + *d*(*w*, *y*, *G*) where *w *is a node on the shortest path identified by the program in running *d*(*x*, *y*, *G*). The idea is that although it is difficult to verify the correctness of each test case (that is, *d*(*x*, *y*, *G*), *d*(*y*, *x*, *G*), *d*(*x*, *w*, *G*) and *d*(*w*, *y*, *G*)), it is much easier to test whether these test cases and their corresponding outputs satisfy the MRs. If the shortest path program violates any of these MRs, we can conclude that the program is incorrect.

### Our contributions

The main contribution of our paper is to introduce the use of MT as a simple, effective and automatable technique for testing bioinformatics programs, particularly for those bioinformatics programs which suffer from the oracle problem. Through two case studies, we give a step-by-step presentation on how MT is used in practice. We explain how to derive MRs from the domain knowledge and program specification, and subsequently how to generate test cases based on these MRs. The effectiveness of MT is demonstrated through analyzing its ability to detect failures in some programs with faults seeded by an automatic process. We also discuss the applicability of MT to various important application domains in bioinformatics.

## Results

The general process of MT is very simple. First, the end user and the tester need to come up with some *properties *involving multiple pairs of inputs and outputs of the target program. These properties are derived from the domain knowledge of the target program. They are referred to as the metamorphic relations (MRs). Second, the tester needs to design a set of source test cases and their corresponding follow-up test cases based on these MRs. In this study, the source test inputs were either generated randomly, or taken from some real-life cases. Finally, all test cases are executed and the outputs of the source and follow-up test cases are compared to check whether the corresponding MR is satisfied. In this section, we demonstrate how MT can be applied in practice through testing of two bioinformatics programs, namely GNLab [14] and SeqMap [15]. In each of our case studies, the intended behaviour of the target program is briefly described, and the process of MR derivation is explained. It should be noted that these two programs are chosen because they are open-source and represent two important application domains in bioinformatics: network simulation and high-throughput data processing.

### Case study 1: Testing of GNLab

GNLab [14,16] is a command-line tool for large-scale analysis and simulation of gene regulatory networks (GRNs). This program consists of separate components for network generation, simulation, analysis, visualization and comparison. Here we only focus on testing the deterministic network simulation component of GNLab. The network simulator of GNLab takes a directed weighted graph as input where the type and strength of gene-to-gene interactions are represented by the edge weights. It assumes the following relationships between the input graph structure and the network dynamics:

1. The weight of an edge represents the influence of the regulator (the node at the tail of the edge) on the target gene (the node pointed by the edge).

2. An edge should have a weight between -1 and 1 inclusively, where a positive weight represents activation and a negative weight represents repression.

3. If two nodes are not connected by a directed path, the expression dynamics of these two nodes should be independent.

This set of GRN simulation rules and previous experience in performing network simulation, which together form the domain knowledge in this case study, indicate that changes in the network structure should cause changes to the execution path of the program. Therefore, we first aim to devise a set of MRs that capture the behaviours of various types of network structure alteration:

#### 1. Addition of a node

(a) **Addition of a disconnected node**. Given a network *G*, we add to it a node *P*, which is not connected to any node in *G*. The new network is referred to as *G'*. The output of *G *should be fully subsumed in the output of *G'*. That is, (the output of *G'*) = (the output of *G*) + (the output of *P*).

(b) **Addition of a non-regulator node**. Given a network *G*, we add to it a node *P*, which is not the regulator to any node in *G*. The new network is referred to as *G'*. The output of *G *should be fully subsumed in the output of *G'*. That is, (the output of *G'*) = (the output of *G*) + (the output of *P*).

#### 2. Addition of an edge

(a) **Addition of an edge with positive weight**. Given a network *G *and a non-regulator node *P *in *G*, we add an edge, which is directed to *P *with a positive weight. The new network is referred to as *G'*. In the output of *G'*, only the output of *P *would be increased, while output of the other nodes should remain unchanged.

(b) **Addition of an edge with negative weight**. Given a network *G *and a non-regulator node *P *in *G*, we add an edge, which is directed to *P *with a negative weight. The new network is referred to as *G'*. In the output of *G'*, only the output of *P *would be decreased, while output of the other nodes should remain unchanged.

(c) **Addition of an edge with zero weight**. Given a network *G *and a node *P *in *G*, we add an edge, which is directed to *P *with zero weight. The new network is referred to as *G'*. The output of *G' *should be identical to the output of *G*.

#### 3. Deletion of a node

(a) **Deletion of a non-regulator node**. Given a network *G *and a non-regulator node *P *in *G*, we delete *P *from *G *to produce a new network *G'*. The output of *G' *should be fully subsumed in the output of *G*. That is, (the output of *G'*) = (the output of *G*) - (the output for *P*).

(b) **Deletion of a regulator node**. Given a network *G *and a regulator node *P *in *G*, we delete *P *from *G *to produce a new network *G'*. Such a deletion should affect the outputs related to all *P'*s *descendant nodes*, while the outputs related to *P'*s *non-descendant nodes *should remain unchanged.

#### 4. Duplication of a network

Given a network *G*, we duplicate *G *to produce a new network *G' *(that is, *G' *= *G *+ *G*). (The output of *G'*) = (the output of *G*) + (the output of *G*).

#### 5. Modification of edge weight

(a) **Increase of edge weight**. Given a network *G*, a node *P *in *G *and an edge *E *directed to *P*, we increase the weight of *E*. Such a modification should increase all the expression values associated with *P*.

(b) **Decrease of edge weight**. Given a network *G*, a node *P *in *G *and an edge *E *directed to *P*, we decrease the weight of *E*. Such a modification should decrease all the expression values associated with *P*.

We used three batches of test cases to test GNLab. The first batch contains some randomly generated input networks as source test cases. The second and third batch of test cases were generated using a yeast GRN [17] and a *E. coli *GRN [18] as source test case respectively. The yeast GRN has 477 nodes and 906 edges, while the *E. coli *GRN has 1306 nodes and 2981 edges [16]. We shall refer to the three batches as batch R (random network), batch Y (yeast GRN) and batch E (*E. coli *GRN). For each of three batches, one pair of source and follow-up test cases were used for each MR (although in general several follow-up test cases can be generated from one source test case, we only used one follow-up test case here to simplify the analysis). All test cases were executed by GNLab and the results were checked against the MRs. The results (see the column under "Original" in Table 1) indicate that GNLab violates MR2(c), while satisfying all other MRs.

**Table 1:** The results of testing GNLab with MT

*MRs*	Original	GM1	GM2	GM3	GM4	GM5	GM6	GM7	GM8	GM9
MR1(a)										
MR1(b)										
MR2(a)				RYE		RYE	RY	Y		
MR2(b)				RYE	RYE		RYE			
MR2(c)	RYE	RYE	RYE	YE	RYE	YE	RYE	RYE	RYE	RYE
MR3(a)										
MR3(b)										
MR4			RYE						RYE	RYE
MR5(a)				RYE	E	RY	RYE	RYE		
MR5(b)				RYE	E	RY	RYE	RYE		

GNLab and nine of its mutants were tested against three batches of test cases, which are labeled as R (random), Y (yeast) and E (*E. coli*). Each pair of test cases that detects a violation of a MR in a program is labeled by its batch in the respective cell in the table. For example, the label 'RY' in the cell [GM6, MR2(a)] indicates that mutant 6 violates MR2(a) according to the test cases in batch R and batch Y.

The initial program developer chose to use *K *= 2000 – 1950|*w*| to map the edge weight of a network into a parameter of the Hill's kinetic equation (see **Methods **for more details). Since the absolute value of the edge weight |*w*| is restricted between 0 and 1, the kinetic parameter *K *is mapped to a value between 2000 and 50. We note that this formula satisfies the intuition that higher weight on a directed edge implies stronger influence of the regulator to the regulated gene (by having smaller *K *value in Hill's equation). However, by using this formula, an edge with zero weight would still allow the regulator (of this zero weight edge) to weakly influence its target gene. To correct this problem, the developer of GNLab either needs to add a conditional clause around this formula, or uses a more appropriate mapping formula. Therefore our results demonstrate the effectiveness of MT by identifying a problem with program specification that leads to a program behaviour which violates a basic network simulation intuition.

### Case study 2: Testing of SeqMap

SeqMap is an efficient tool for mapping massive amount of short sequence reads to a reference genome [15]. One important application of SeqMap is the detection of cross-hybridizing probes in an oligonucleotide microarray [19]. SeqMap performs short sequence mapping by solving an approximate string matching problem, which is defined as follows: Given a reference string *p *and a set of target strings *T *= {*t*_1_, *t*_2_, ..., *t*_*k*_} where all strings are made up of a finite number of characters taken from the set of alphabets *A *= {*a*_1_, *a*_2_, ..., *a*_*m*_}, the task is to find all substrings in *p *such that each substring has an edit distance equal to or less than the maximum number of mismatches *e *against each *t*_*i *_∈ *T*. Edit distance refers to the number of operations required to transform one string to another. In short sequence mapping (SSM), *p *is the genome and *T *is the set of short sequence reads which we intend to map to the genome. In particular, the set of allowable alphabets is *A *= {A, T, G, C}, and valid edit operations are substitution, insertion and deletion. If a sequence read *t*_*i *_matches any substring in genome *p*, it is said to be *mappable*, otherwise, it is said to be *unmappable*.

Although it is easy to verify whether the mapping of each read at one genome location has a mismatch number that is equal to or less than the maximum number of mismatches, it is very hard to check whether this read has been mapped to all possible matching positions in the genome. Further it is also very hard to check whether all unmappable reads are indeed truly unmappable to the genome. In other words, soundness of the result is easy to verify, but not the completeness of the result. Therefore testing of SeqMap suffers from the oracle problem, and may benefit from MT.

Based on the knowledge of the problem domain, we have identified two important variables that most likely affect the execution pattern of the program: the content of the input sequences, both *p *and *T*, and the edit distance constraint, *e*. We therefore derived a set of MRs based on the properties of these variables:

#### 1. Changes in read of *T*

(a) **Addition of mismatches**. Given a set of sequence reads *T *= {*t*_1_, *t*_2_, ..., *t*_*n*_}, a genome *p *and a maximum number of mismatches *e*, we map *T *to *p *and denote the set of mappable reads as *T*_*m *_= $\left\{{t^{\prime }}_{1},{t^{\prime }}_{2},...,{t^{\prime }}_{k}\right\}$, where *k *≤ *n*. Define a subset *M *of *T*_*m*_, where *M *= {*m*_1_, *m*_2_, ..., *m*_*q*_} and *q *≤ *k*. For any *m*_*i *_∈ *M*, assume *l *as one of its mismatch numbers. We arbitrarily choose a *l'*, such that *l *<*l' *≤ *e*, and introduce (*l' *- *l*) new mismatches on *m*_*i *_(denoted as ${m^{\prime }}_{i}$). Then ${m^{\prime }}_{i}$ should still be mappable to *p*. Furthermore, there should exist at least one common location for *m*_*i *_and ${m^{\prime }}_{i}$.

(b) **Removal of mismatches**. Given a set of sequence reads *T *= {*t*_1_, *t*_2_, ..., *t*_*n*_}, a genome *p *and a maximum number of mismatches *e*, we map *T *to *p *and denote the set of mappable reads as *T*_*m *_= $\left\{{t^{\prime }}_{1},{t^{\prime }}_{2},...,{t^{\prime }}_{k}\right\}$, where *k *≤ *n*. Define a subset *M *of *T*_*m*_, where *M *= {*m*_1_, *m*_2_, ..., *m*_*q*_} and *q *≤ *k*. For any *m*_*i *_∈ *M*, assume *l *as one of its mismatch numbers. We arbitrarily choose a *l'*, such that 0 ≤ *l' *<*l*, and delete (*l *- *l'*) mismatches on *m*_*i *_(denoted as ${m^{\prime }}_{i}$). Then ${m^{\prime }}_{i}$ should still be mappable to *p*. Furthermore, there should exist at least one common location for *m*_*i *_and ${m^{\prime }}_{i}$.

(c) **Change the type of mismatch**. Given a set of sequence reads *T *= {*t*_1_, *t*_2_, ..., *t*_*n*_}, a genome *p *and a maximum number of mismatches *e*, we map *T *to *p *and denote the set of mappable reads as *T*_*m *_= $\left\{{t^{\prime }}_{1},{t^{\prime }}_{2},...,{t^{\prime }}_{k}\right\}$, where *k *≤ *n*. Define a subset *M *of *T*_*m*_, where *M *= {*m*_1_, *m*_2_, ..., *m*_*q*_} and *q *≤ *k*. For any *m*_*i *_∈ *M*, assume *l *as one of its mismatch numbers. We arbitrarily replace several mismatches by different types of mismatch on *m*_*i *_(denoted as ${m^{\prime }}_{i}$), while keeping the same total number of mismatches as *l *(for example, a substitution can be replaced by a deletion without affecting the number of mismatches). Then ${m^{\prime }}_{i}$ should still be mappable to *p*. Furthermore, there should exist at least one common location for *m*_*i *_and ${m^{\prime }}_{i}$.

#### 2. Changes in *p*

(a) **Concatenation of subset of reads**. Given a set of sequence reads *T *= {*t*_1_, *t*_2_, ..., *t*_*n*_}, and a genome *p*, we select a subset of sequence reads *TS *⊂ *T *and concatenate this subset of reads to the end of *p *to form a new genome *p'*. After mapping *T *to both *p *and *p' *independently, the following relations should hold: (1) all reads in *T *that are mappable to *p *should also be mappable to *p'*, and (2) each read in *TS *that is mappable to *p *should have at least one additional mapping location in the part of *p'*, which corresponds to the concatenated string. (3) each read in *TS *that is unmappable to *p *should be mapped at least once in the part of *p'*, which corresponds to the concatenated string.

(b) **Deletion of ***p*. Given a set of sequence reads *T *= {*t*_1_, *t*_2_, ..., *t*_*n*_}, and a genome *p*, we form a new genome *p' *by deleting an arbitrary portion of either the beginning or ending of *p*. After mapping *T *to both *p *and *p' *independently, all reads in *T *that are unmappable to *p *should also be unmappable to *p'*.

#### 3. Changes in both *p *and *T*

(a) **Reversing the input ***p ***and ***T*. Given a set of sequence reads *T *= {*t*_1_, *t*_2_, ..., *t*_*n*_}, and a genome *p*, we form a new set of sequence reads *T' *= $\left\{{t^{\prime }}_{1},{t^{\prime }}_{2},...,{t^{\prime }}_{n}\right\}$ and *p' *such that each string ${t^{\prime }}_{i}$ is a reversed string of *t*_*i *_for 1 ≤ *i *≤ *n*, and *p' *is a reversed string of *p*. A string *s' *is a reversed string of *s *if the first character of *s' *is the last character of *s *and the second character of *s' *is the second last character of *s*, and so on. We map *T *to *p *and independently map *T' *to *p'*. The following relations should hold: (1) *t*_*i *_is mappable to *p *if and only if ${t^{\prime }}_{i}$ is mappable to *p' *for 1 ≤ *i *≤ *n*, and (2) *t*_*i *_is unmappable to *p *if and only if ${t^{\prime }}_{i}$ is unmappable to *p' *for 1 ≤ *i *≤ *n*.

(b) **Permutation of alphabets**. Given a set of sequence reads *T *= {*t*_1_, *t*_2_, ..., *t*_*n*_}, a genome *p*, and a one-to-one permutation function on the set of alphabets, *Permute*. For any string *s*, *Permute*(*s*) is used to denote the string after permutation. We define a new set of sequence reads *T' *= $\left\{{t^{\prime }}_{1},{t^{\prime }}_{2},...,{t^{\prime }}_{n}\right\}$ and *p' *such that ${t^{\prime }}_{i}$ = *Permute*(*t*_*i*_) for all 1 ≤ *i *≤ *n*, and *p' *= *Permute*(*p*). We map *T *to *p *and independently map *T' *to *p'*. The following relations should hold: (1) *t*_*i *_is mappable to *p *if and only if ${t^{\prime }}_{i}$ is mappable to *p' *for 1 ≤ *i *≤ *n*, and (2) *t*_*i *_is unmappable to *p *if and only if ${t^{\prime }}_{i}$ is unmappable to *p' *for 1 ≤ *i *≤ *n *.

#### 4. Changes in maximum number of mismatches (*e*)

(a) **Decrease of ***e*. Given a set of sequence reads *T *= {*t*_1_, *t*_2_, ..., *t*_*n*_}, a genome *p*, and the maximum number of mismatches *e*, we create a new *e' *such that 0 ≤ *e' *<*e*. We map *T *to *p *with parameter *e *and denote the set of mappable reads as *M*. We independently map *T *to *p *with parameter *e' *and denote the set of mappable reads as *M'*. The following relation should hold: *M' *⊆ *M*.

(b) **Increase of ***e*. Given a set of sequence reads *T *= {*t*_1_, *t*_2_, ..., *t*_*n*_}, a genome *p*, and the maximum number of mismatches *e*, we create a new *e' *such that 0 ≤ *e *<*e'*. We map *T *to *p *with parameter *e *and denote the set of mappable reads as *M*. We independently map *T *to *p *with parameter *e' *and denote the set of mappable reads as *M'*. The following relation should hold: *M *⊆ *M'*.

Using MT, we tested SeqMap (version 1.0.8) with five batches of test cases. For each batch, we randomly generated a source test case, from which one follow-up test case is constructed based on each MR. The execution results were checked against these MRs, and we did not observe violation to any of them.

### Mutation analysis of GNLab and SeqMap

To better demonstrate the applicability of MT, we generated a number of fault-seeded variants of GNLab and SeqMap, and measure how well our MT procedure can detect them. This process is referred to as *mutation analysis *[20,21]. A program with a seeded fault is called a *mutant*. All mutants are generated by applying some very simple *mutation operators *to alter the source code of the original program. Previous study shows that, despite the simplicity of these mutation operators, the capability of detecting failures from the generated mutants is a good indicator of the effectiveness of a testing method [20]. It should be noted that if a mutant produces the same output as the original program for all possible inputs with respect to the functionalities under test, such a mutant is said to be *equivalent *to the original program. Using an unbiased random mutant generation strategy (as described in the **Methods **section), we initially generated 25 mutants for GNLab and 20 mutants for SeqMap. Since we only focus on testing certain functionalities of GNLab and SeqMap (namely, deterministic network simulation and approximate sequence matching, respectively), many of the automatically mutated statements in the source code do not affect the targeted functionalities, and therefore many mutants are equivalent mutants. As a result, there are nine non-equivalent mutants for GNLab and three non-equivalent mutants for SeqMap. Although the pool of applicable (non-equivalent) mutants is not very large, the results we obtain here is sufficient for our purpose of demonstrating the applicability of MT in bioinformatics.

The nine non-equivalent mutants of GNLab (denoted by GM1, GM2, ..., and GM9) were tested by three batches of test cases used in the testing of the original GNLab program. The results are shown in Table 1. We observe that all mutants violate at least one MR. It is also worth noting that different MRs are effective in revealing the fault in different mutants. Moreover, different MRs have different failure-detection capabilities. Test cases associated with MRs 1(a), 1(b), 3(a) and 3(b) cannot detect any failure. In other words, all the MRs related to the modification of nodes in GNLab network are less effective than other kinds of MRs. The MR violation rate (the proportion of pairs of source and follow-up test cases that violate a MR) of the three batches are: 0.267 (R), 0.3 (Y), and 0.278 (E). Further, for a given mutant, a single batch of test cases may not violate all possible MRs. For example, only batch E can reveal a violation of MR5(a) and (b) in GM4, and only batch Y can reveal a violation of MR2(a) in GM7. This shows the importance of using various test cases when conducting testing.

The three non-equivalent mutants of SeqMap (denoted by SM8, SM11 and SM18) were tested by the same five batches of test cases used in testing the original SeqMap program. The results (shown in Table 2) shows that our test cases can effectively detect failures in all three mutants. Similar to the testing of GNLab, we observe that different sets of MRs are being violated when testing the same mutant with different test cases. Also, we note that for each mutant, at least one MR is violated by the test cases.

**Table 2:** The results of testing SeqMap with MT

*MRs*	Original	SM8	SM11	SM18
MR1(a)		1,2,3,4,5	1,2,3,4,5	1,4,5
MR1(b)		1,2,3	1,2,3,4,5	5
MR1(c)		1,2,3,4,5	1,2,3,4,5	1,4
MR2(a)			1,2,3,4,5	1,2,3,4,5
MR2(b)			1,2,3,4,5	1,2,5
MR3(a)		1,2,3,4,5	1,2,3,4,5	1,4,5
MR3(b)		1,3,4,5	1,2,3,4,5	
MR4(a)		2,3,4,5	1,2,3,4,5	1,2,3,4,5
MR4(b)		1,2,3,4,5	1,2,3,4,5	

SeqMap and three of its mutants were tested against five batches of test cases, which are labeled as 1, 2, 3, 4 and 5. Each pair of test cases that detects a violation of a MR in a program is labeled by its batch in the respective cell in the table. For example, the label '1,4' in the cell [SM18, MR1(c)] indicates that SM18 violates MR1(c) according to the test cases in batch 1 and batch 4.

## Discussion

### Applicability of metamorphic testing in bioinformatics

#### MT is a general technique to alleviate the oracle problem

The programs we used in our case studies belong to two types of bioinformatics programs that are traditionally very hard to test due to the lack of a tangible oracle. For instance, the current approach to test a network simulator involves visual inspection of the simulated values as well as comparison among multiple implementations of a simulator [7]. Therefore it tries to tackle the oracle problem by using multiple implementations. Such multiple implementations are often hard to acquire in practice, and the results of such testing may be hard to interpret when different implementations give different results. Our approach for alleviating the oracle problem is through verifying relationships among multiple test cases. As demonstrated by both of our case studies, as well as many previous studies [5,6,11,22], MT is effective for testing programs that are traditionally difficult to test due to the oracle problem. This allows MT to be applicable for testing various bioinformatics programs in which the oracle problem exists.

#### MT can test a program against its intended behaviour

For any program, MRs can be derived from the intended program behaviour or the program specification. As demonstrated in our case studies, all MRs are based on the intended program behaviour (that is, from the domain knowledge of network dynamics and approximate string matching), and they do not make use of the details of the implementations (for example, the underlying algorithm and data structure). Based on the ten MRs derived from the intended behaviour of a GRN simulator, we found a fault in GNLab. As explained in the **Results **section, this fault is due to the mis-specification of algorithm instead of a bug in the implementation. This means, if test cases were derived from the specification alone, this fault may not have been detected. We believe this ability to test a program against its intended behaviours is very important in bioinformatics, as it allows us to focus our testing effort on assessing whether the underlying biological questions are being tackled correctly. Of course, we can easily derive MRs from program specification as well.

#### MT can be combined with special test cases

It should be emphasized that MT is a general testing technique for the situations where there is no tangible oracle. It can be used to generate a large amount of test cases based on an existing set of test cases. For example, we can easily construct some artificial short sequence reads with predefined mismatch patterns for the testing of SeqMap. Such special test cases are useful and should be used as far as possible. However, special test cases only cover a small portion of all possible inputs, and we still need more test cases whose testing results are not easy to verify. A straightforward method is to combine MT technique with special test cases. This can be done by using each special test case as a source test case to generate follow-up test cases based on some MRs. Such an approach has been shown to be very effective in detecting non-trivial faults [5].

#### MT is simple and automatable

As demonstrated through the two case studies, the process of MT is straightforward. Different subsets of behaviours of the target program can be tested by employing different MRs. Once the MRs are identified, test cases can easily be automatically generated and their outputs can be verified using simple scripts. A single program can have a great number of MRs, and various follow-up test cases can be defined based on one single source test case. Moreover, the simplicity of MT allows us to perform systematic automated testing using a simple test script. The use of simple test script is important to minimize the chance of introducing bugs into the test script itself, which can subsequently confound the interpretation of the testing result.

#### MT allows the use of real inputs as test cases

One implication of the ability to automatically generate more test cases is that we can now use real-life program inputs as test inputs. In the MT framework, it is easy to treat a real-life input as a source test case, and generate many follow-up test cases using a set of MRs. For programs that lack a tangible oracle, test cases are usually restricted to those that can easily be constructed and verified. Such test cases may not have the same size and characteristics as the real-life program inputs. For instance, testing of real-life input is often not possible for most network simulators since we have no objective means to verify the large amount of simulation results. Using MT, such difficulty is alleviated by testing the outputs against a set of MRs instead of the oracle. In our case study of GNLab, we can construct test cases based on two real-life GRNs which are much larger and complicated than the randomly generated ones. Many bioinformatics programs deal with high-throughput data, therefore the ability to test whether they can correctly handle such real-life inputs is important.

#### MT is suitable for bioinformatics programmers

Compared to many other testing techniques, MT is much easier to implement in practice because it relies mainly on user domain knowledge rather than software testing knowledge. Many bioinformatics programs are developed by the end-user – the researcher or research group who uses this program. Chen *et al*. [13] have demonstrated that MT is particularly suited to test end-user programmers' own programs because (1) "end-user programmers have the domain knowledge to identify MRs" and (2) "end-user programmers can distinguish good MRs based on program structures" [13]. In the GNLab example, we only identified and used some MRs related to the structure of the input networks because our domain knowledge points out that changing the network structure should induce the most changes in the execution of the simulator. Other properties related to the execution of the simulation, such as length of simulation and output interval, are not covered by the MRs. This feature of MT allows the tester to focus most of the testing effort on the subset of functionalities that are more important, or more frequently used by its intended users.

#### MT is useful for testing diverse types of programs

Although both programs used in our case studies implement deterministic procedures, some initial results show that MT can also be used for testing other types of procedure that are traditionally difficult to test, such as heuristic methods, machine learning methods, stochastic methods and so on [23,24]. Since many bioinformatics programs implement such procedures, we expect MT to be applicable to them.

### Limitations

It should be noted that satisfying all test cases based on a set of MRs does not guarantee the correctness of the program under test. MRs are necessary properties, hence satisfying all of them is not sufficient to guarantee program correctness. This problem is, in fact, a limitation of all software testing methods. Nonetheless, the ability to systematically produce a large number of test cases should increase our chance of detecting a fault in the target program, and hence improve its quality.

As this paper focuses on introducing the application of MT in bioinformatics, there are other issues related to MT that are not explicitly discussed here. First, the success of MT greatly depends on defining a "good" set of MRs. From the testing results of GNLab and SeqMap, we observe that some MRs are less effective in detecting faults than others. In particular, we note that all MRs based on adding and removing nodes from a network is not effective in detecting faults in our fault-seeded mutants of GNLab. So far in this paper, we have not explicitly addressed the issues of selecting effective MRs. Some initial results suggest that those MRs which trigger different execution paths for the source and follow-up cases are more likely to reveal faults [25]. This means, although deriving MRs is usually straightforward, selecting the most effective MRs requires good understanding of the problem domains. More specific guidelines in choosing MRs is being actively investigated. Second, the MT technique itself does not specify how source test cases should be selected given a set of MRs. We have used randomly generated inputs and real-life inputs for generating source test cases in our study. However, as shown in our case studies, the performance of MT also depends on the number and variety of source test cases. We expect that MT can be combined with other established test case selection techniques to improve the fault-revealing ability.

### Further examples in bioinformatics

Beside programs for network simulation and short sequence mapping, we notice that many other bioinformatics programs can benefit from MT. Here we briefly discuss how the testing of programs from several important bioinformatics domains suffer from the oracle problem, and how MT technique can be used in each case. The list of applications presented here is by no means exhaustive. Only very simple MRs are pointed out here as we are not discussing any particular detailed problem description. In general, more complex, and potentially more fault revealing, MRs can be formulated based on a more thorough understanding of the problem domain or program specification [25].

#### Phylogenetics

One major endeavor in phylogenetics is to infer the phylogeny (phylogenetic tree) of some species based on their aligned nucleotide or amino acid sequences [26]. There are three main approaches to phylogenetic inference: (1) parsimony methods, (2) distance based methods, (3) model based methods. Broadly speaking, all methods aim to group these species into a binary tree according to different measures of sequence relatedness. We commonly analyze large number of long bio-sequences. Also, many of these methods involve calculating distance matrix, or computing maximum-likelihood estimates, which are difficult to verify except for trivial inputs. Therefore the testing of phylogenetic inference programs suffers from the oracle problem.

Let us denote a program that performs phylogenetic inference as *P*. The input of *P *is a set of *n *aligned sequences **S **= {*S*_1_, *S*_2_, ..., *S*_*n*_}, and the output is a binary tree *T*(**S**). One possible MR is that adding a sequence, *S*_*n*+1_, would not change the relative structure of the rest of the tree. That is, the trees generated by the source case *T*(**S**) and the follow-up case *T*(**S **∪ *S*_*n*+1_) only differ by one additional leaf node representing *S*_*n*+1_. For a *P *that treats each alphabet independently and equally, we can define another MR: replacing, or permuting, the alphabet of the sequence with one another (for example, A ↦ C, T ↦ A, G ↦ T, C ↦ G) does not change the final structure of the tree. That is, *T*(**S**) = *T*(Permute(**S**)) where Permute( ) is an alphabet permutation function.

#### Microarray analysis

Microarray analysis has become an indispensable tool in modern biological and medical research. Many types of analyses are available for analyzing microarray data. They include differential expression (DE) [27], differential variability (DV) [28], hierarchical clustering [29], gene set enrichment analysis [30] and Bayesian network analysis [31]. Due to the difficulty in analyzing the high dimensional input (microarray expression profiles), and often also the high dimensional output (ranked gene list, binary tree, and Bayesian network), the correctness of the implementation is often difficult to verify. In this case, MT technique can be useful. Let us take the identification of DE genes between two sample classes as an example. One simple approach is to use the *t*-statistics to obtain a *P *value for each gene based on a two-sided hypothesis, and call the genes with *P *less than a pre-specified threshold significant DE genes. Since *t*-statistics is shift independent, we can define a MR that adding a constant value to all values in the input microarray profile does not alter the resulting list of *P *values. The second MR is that switching the class label of the samples also does not alter the resulting *P *values as the *t*-distribution is symmetrical.

#### Biological database retrieval

Many biomolecular databases are available, and most of them are built to support fast data retrieval and database mining [18,32-34]. One major challenge is to ensure that we can accurately and efficiently retrieve the desired data item from the database. This is particularly important as we begin to construct large scale gene regulatory networks and metabolic networks using these databases. Invalid retrieval results may lead to a false positive or false negative edges in a reconstructed network. Due to large size of the database, it is generally difficult to test if a search engine can correctly retrieve all data that exactly match a query. A potentially suitable MR is that a query *A *∩ *B *∩ *C *should not contain more results than query *A *∩ *B*. Another MR is that executing the query ¬(*A *∪ *B*) should have the same effect as executing the query (¬*A*) ∩ (¬*B*). Many more MRs along this line are possible.

## Conclusion

Issues related to proper software testing have been largely overlooked in the bioinformatics community. As discussed in this paper, systematic testing of many bioinformatics programs is difficult due to the oracle problem, that is, it is very difficult to verify the testing results of these programs. In this paper, we propose to apply an innovative software testing technique, metamorphic testing (MT), to test these bioinformatics programs. As a case study, we applied the MT technique to test a network simulator and a short sequence mapping program. The results demonstrate that MT is simple to implement, and is effective in revealing faults in a program.

We believe that our work have significant contributions. As far as we are aware, this is the first paper that systematically discusses the oracle problem in testing bioinformatics programs, and how it can be alleviated. As biologists increasingly rely on the results produced by these bioinformatics programs, it is crucial to ensure that they are of high quality. We wish this paper can raise the awareness of proper software testing practice in the bioinformatics community.

## Methods

### Specification of GNLab

For the simulation of expression dynamics, GNLab [14] models a GRN as a system of ordinary differential equations (ODEs) using Hill's kinetics [35,36]. In particular, Mendes *et.al*.'s multiplicative model [37] is used. Assume that *X*(*G*_*i*_) represents the level of mRNA of gene *G*_*i*_, and it is activated by genes $G_{a_{1}},G_{a_{2}},...,G_{a_{m}}$ and repressed by genes $G_{r_{1}},G_{r_{2}},...,G_{r_{m}}$. Constants $K_{a_{i}}$ and $K_{r_{j}}$ represent the expression levels of $G_{a_{i}}$ and $G_{r_{j}}$ respectively at which the effect on the target gene is half of its saturating value. The Hill's constant *n *controls the sigmoidicity of the interaction curve. *V*_*i *_represents the basal transcriptional rate of gene *G*_*i*_. The rate law for mRNA synthesis can therefore be formulated as:

(1) $$syn(G_{i})=V_{i}\prod_{i=1}^{m}(1+\frac{X{\left(G_{a_{i}}\right)}^{n}}{X{\left(G_{a_{i}}\right)}^{n}+K_{a_{i}}^{n}})\prod_{j=1}^{n}\frac{K_{r_{j}}^{n}}{X{\left(G_{r_{j}}\right)}^{n}+K_{r_{j}}^{n}}$$

The rate mRNA degradation is assumed to only depend linearly on the current expression level, that is,

(2) *break*(*G*_*i*_) = *b*·*X*(*G*_*i*_)

Overall, the change of gene expression level for a gene *G*_*i *_is modeled by the following ODE:

(3) $$\frac{dG_{i}}{dt}=syn(G_{i})-break(G_{i})$$

Based on the intended behaviours of GNLab, a graph is converted to a collection of ODEs where each node in the graph represents a gene and its associated ODE is dependent on the incoming edges. The weight of each edge is converted to a number of kinetic parameters in the ODE model as described in Equations 1 and 2 (including *K*, *V*, *n *and *b*). GNLab uses the following default parameter settings: *V *= 10, *b *= 0.01, and *n *= 4. The relationship between arc weight *w *(where 0 ≤ |*w*| ≤ 1) and interaction strength *K *is encapsulated in the function *K *= 2000 – 1950|*w*|. The initial value for all *G*_*i *_is set to be 50. The set of ODEs is simulated by the Euler method.

### Specification of SeqMap

To achieve the goal of efficient short sequence read mapping, SeqMap [15] first builds an index of all the short sequence reads. The genome is then scanned to find candidate short sequence reads that can match to each position. In particular, SeqMap uses a two-phase strategy to speed up the matching process. In the first phase, the short sequences are split into several parts given the allowed edit distance constraints. Since the allowable mismatches can only occur at some parts of a sequence, SeqMap can quickly eliminate some non-candidate by the pigeon-hole principle. In the second phase, a local alignment algorithm is run to determine the matching target.

### Execution of GNLab and SeqMap

For GNLab, all test cases were executed by the command 'GNLab -t input d 150 0.1 150', which specifies that the network input.gnl.txt is to be simulated by a deterministic simulator with 150 output display using Euler method with step-size of 0.1 and with no perturbation during the simulation. For SeqMap, most test cases are executed by the command 'seqmap 3 T.fa P.fa out.txt /allow_insdel:3 /output_alignment /silent /output_all_matches /output_top_matches:6', which maps short sequence reads in file T.fa to a reference genome in file P.fa with a maximum of 3 mismatches allowed. The maximum number of mismatches may change for testing MR4(a) and 4(b).

### Mutation analysis

To generate mutants for GNLab and SeqMap, we applied four popular mutation operators to the source code of the two programs: arithmetic operator replacement (AOR), constant replacement (CR), relational operator replacement (ROR), and scalar variable replacement (SVR). In order to avoid any potential bias, the fault-seeding process is not manually decided by a tester but randomly selected by the following two steps: First, a program statement is randomly selected as the *target statement *for modification. Then, a mutation operator is randomly chosen to apply to this target statement to generate a mutant, with the constraint that this modification yields a syntactically correct statement. For example, the statement if(x>0) may be changed to if(x<0) by a ROR operator. Using this strategy, we generated 25 mutants for GNLab and 20 mutants for SeqMap. Among these mutants, there are nine mutants for GNLab and three mutants for SeqMap that are not equivalent to their respective original program in terms of the investigated functionalities. We only used these non-equivalent mutants as the target programs in our study.

In particular, the process of selecting target statements to be mutated is different between GNLab and SeqMap. All statements in the source code of SeqMap can be selected to become the target statement for mutation, but only a portion of the complete GNLab source code could be selected as target statements for mutation. This is because GNLab is a large program that contains many other additional functionalities (such as network analysis and stochastic network simulation), so we decided to exclude those statements that are definitely not related to the deterministic simulator to avoid excessive number of equivalent mutants. Such a difference contributed to the apparent difference between the non-equivalent mutant generation rate: 3/20 for SeqMap and 9/25 for GNLab. However, as described in the **Results **section, this set of mutants is sufficient for the purpose of our study.

## Authors' contributions

TYC and JWKH conceived and initiated the project. All authors contributed to the overall design and implementation of the experiment. HL, XX and JWKH conducted the experiments and analyzed the results. JWKH and HL wrote the first draft of the manuscript. All authors contributed to, read, and approved the final version of this manuscript.
